# Parasitaemia data and molecular characterization of *Haemoproteus catharti* from New World vultures (Cathartidae) reveals a novel clade of *Haemosporida*

**DOI:** 10.1186/s12936-017-2165-5

**Published:** 2018-01-08

**Authors:** Michael J. Yabsley, Ralph E. T. Vanstreels, Ellen S. Martinsen, Alexandra G. Wickson, Amanda E. Holland, Sonia M. Hernandez, Alec T. Thompson, Susan L. Perkins, Christopher J. West, A. Lawrence Bryan, Christopher A. Cleveland, Emily Jolly, Justin D. Brown, Dave McRuer, Shannon Behmke, James C. Beasley

**Affiliations:** 1 Warnell School of Forestry and Natural Resources, The University of Georgia, Athens, GA USA; 2 Southeastern Cooperative Wildlife Disease Study, Department of Population Health, College of Veterinary Medicine, The University of Georgia, Athens, GA USA; 3 Marine Apex Predator Research Unit, Institute for Coastal and Marine Research, Nelson Mandela University, Port Elizabeth, South Africa; 4 DST/NRF Centre of Excellence at the Percy FitzPatrick Institute, Department of Zoology, Nelson Mandela University, Port Elizabeth, South Africa; 5 Center for Conservation and Evolutionary Genetics, Smithsonian Conservation Biology Institute, National Zoological Park, Washington DC, USA; 6 Department of Biology, University of Vermont, Burlington, VT USA; 7 Savannah River Ecology Laboratory, University of Georgia, Aiken, SC USA; 8 Sackler Institute for Comparative Genomics, American Museum of Natural History, New York, NY USA; 9 Yurok Tribe Wildlife Program, Klamath, CA USA; 10 Pennsylvania Game Commission, Animal Diagnostic Laboratory, University Park, PA USA; 11 Wildlife Center of Virginia, Waynesboro, VA USA; 12 Davis College of Agriculture, Natural Resources and Design, Division of Forestry and Natural Resources, West Virginia University, Morgantown, WV USA

**Keywords:** Adenylosuccinate lyase, Avian parasite, Bird, Cytochrome b, Cathartidae, Evolution, Haemoproteidae, Malarial parasite, North America, Neotropical

## Abstract

**Background:**

New World vultures (Cathartiformes: Cathartidae) are obligate scavengers comprised of seven species in five genera throughout the Americas. Of these, turkey vultures (*Cathartes aura*) and black vultures (*Coragyps atratus*) are the most widespread and, although ecologically similar, have evolved differences in morphology, physiology, and behaviour. Three species of haemosporidians have been reported in New World vultures to date: *Haemoproteus catharti*, *Leucocytozoon toddi* and *Plasmodium elongatum*, although few studies have investigated haemosporidian parasites in this important group of species. In this study, morphological and molecular methods were used to investigate the epidemiology and molecular biology of haemosporidian parasites of New World vultures in North America.

**Methods:**

Blood and/or tissue samples were obtained from 162 turkey vultures and 95 black vultures in six states of the USA. Parasites were identified based on their morphology in blood smears, and sequences of the mitochondrial cytochrome b and nuclear adenylosuccinate lyase genes were obtained for molecular characterization.

**Results:**

No parasites were detected in black vultures, whereas 24% of turkey vultures across all sampling locations were positive for *H. catharti* by blood smear analysis and/or PCR testing. The phylogenetic analysis of cytochrome b gene sequences revealed that *H. catharti* is closely related to MYCAMH1, a yet unidentified haemosporidian from wood storks (*Mycteria americana*) in southeastern USA and northern Brazil. *Haemoproteus catharti* and MYCAMH1 represent a clade that is unmistakably separate from all other *Haemoproteus* spp., being most closely related to *Haemocystidium* spp. from reptiles and to *Plasmodium* spp. from birds and reptiles.

**Conclusions:**

*Haemoproteus catharti* is a widely-distributed parasite of turkey vultures in North America that is evolutionarily distinct from other haemosporidian parasites. These results reveal that the genetic diversity and evolutionary relationships of avian haemosporidians are still being uncovered, and future studies combining a comprehensive evaluation of morphological and life cycle characteristics with the analysis of multiple nuclear and mitochondrial genes will be useful to redefine the genus boundaries of these parasites and to re-evaluate the relationships amongst haemosporidians of birds, reptiles and mammals.

**Electronic supplementary material:**

The online version of this article (10.1186/s12936-017-2165-5) contains supplementary material, which is available to authorized users.

## Background

The Order *Haemosporida* contains numerous vector-borne protozoan blood parasites of reptilian, avian and mammalian hosts [1, 2]. These parasites utilize a wide range of vectors and are found on all continents except Antarctica. There are numerous genera of haemosporidian parasites, four of which have species recorded in avian hosts: *Plasmodium* (described in 1885), *Haemoproteus* (1890), *Leucocytozoon* (1904) and *Fallisia* (1974) [2]. Considering genetic evidence and differences in natural history, however, many researchers have suggested that the two subgenera of *Haemoproteus* (*Haemoproteus* and *Parahaemoproteus*) and the two subgenera of *Leucocytozoon* (*Akiba* and *Leucocytozoon*) should be elevated to genera [3, 4]. A recent study has also uncovered genetic evidence that indicates *Haemoproteus antigonis*, a parasite from cranes (Gruidae), represents a novel clade that is paraphyletic with other known *Haemosporida*, potentially meriting a separate genus [5].

Despite the vast amount of information on the haemosporidians of birds, knowledge about the species that infect vultures is still very limited. Vultures are large, obligate scavenging birds that are divided into two families, the Old World vultures (Accipitridae: Aegypiinae, Gypaetinae) and the New World vultures (Cathartidae). The morphological and physiological similarities between these groups are a remarkable example of convergent evolution [6, 7]. There are currently seven species of New World vultures in five genera, four of which are monotypic [8]. Of these, turkey vultures (*Cathartes aura*, TUVU) and black vultures (*Coragyps atratus*, BLVU) are the most widespread, ranging from southern South America into the United States, and even Canada in the case of turkey vultures. Despite similarities in appearance and functional role in ecosystems, black and turkey vultures have evolved unique morphological, physiological, and behavioural differences that result in interspecific differences in foraging behaviour [9, 10]. With the exception of the California condor (*Gymnogyps californianus*), which has a restricted distribution in the western United States, the remaining New World vulture species reside in Central and South America [8].

Four species of haemosporidians have been reported in Old World vultures, *Haemoproteus elani*, *Haemoproteus janovyi*, *Leucocytozoon toddi* and *Plasmodium fallax* [11–14], whereas three species have been recorded in New World vultures, *Haemoproteus catharti*, *Leucocytozoon toddi* and *Plasmodium elongatum* [15, 16]. Additionally, there are numerous records of *Haemoproteus* sp., *Plasmodium* sp. and *Leucocytozoon* sp. in New World vultures that have not been morphologically or genetically characterized (Table 1). Currently, the only publicly-available sequence of a haemosporidian parasite of New World vultures corresponds to a *Plasmodium* sp. lineage NYCNYC01, which was detected in a captive King Vulture (*Sarcoramphus papa*) from São Paulo Zoo, Brazil [17].

**Table 1:** Published records of hemosporidian parasites in New World vultures

Species	Parasite	Location	Apparent prevalence	References
Turkey vulture (TUVU)	*Haemoproteus* sp.^a^	Panama	Not reported	[41]
	*Haemosporida* ^b^	USA (Maryland)	1	[16]
	*Haemoproteus* sp.	USA (Washington DC)	14/79 (18)	[42]
	*Leucocytozoon* sp.	USA (Washington DC)	2/79 (3)	[42]
	*Haemoproteus* sp.	USA (Georgia)	1/4 (25)	[43]
	*Haemoproteus* sp.	Panama	3/4 (75)	[32]
	*Haemoproteus* sp.	USA (Maryland and New Jersey)	2/9 (22)	[44]
	*Haemoproteus catharti* and *Plasmodium* sp.^c^	USA (South Carolina)	1/11 (9)	[16, 45]
Black vulture (BLVU)	*Haemoproteus* sp.	USA (Oklahoma)	1/1 (100)	[12]
	*Plasmodium elongatum* and *Leucocytozoon toddi*	USA (Florida)	1/211 (0.5)	[15]
	*Haemosporida* ^d^	Costa Rica	6/17 (35)	[46]
King vulture	*Plasmodium* sp.	Brazil (São Paulo)	1	[17]

Apparent prevalence is shown as: number of samples positive/number of samples tested (%)

^a^Originally identified as *H. danilewskii*, this record was later revised as *Haemoproteus* sp. [45]

^b^Slides deposited in the International Reference Centre for Avian Haematozoa (Queensland Museum, Australia) were re-examined and found to correspond to an haemosporidian that was distinct from *H. catharti*, possibly corresponding to *P. elongatum* [16]

^c^A small number of immature *Plasmodium* sp. schizonts (possibly *P. circumflexum* or *P. galbadoni*) was seen concurrently with *H. catharti*

^d^These parasites were reported as *Plasmodium* sp., however images of the parasites provided by M. Wahl were reviewed and only very young gametocytes were observed, therefore parasite genus cannot be determined

In this study, morphological and molecular methods were used to investigate the epidemiology and evolution of haemosporidian parasites of New World vultures sampled at six states of the USA. These data challenge the placement of *H. catharti* in the genus *Haemoproteus*, and instead suggest that these parasites represent a novel evolutionary lineage of haemosporidians, possibly meriting a separate genus.

## Methods

### Sample collection

**Fig. 1:**
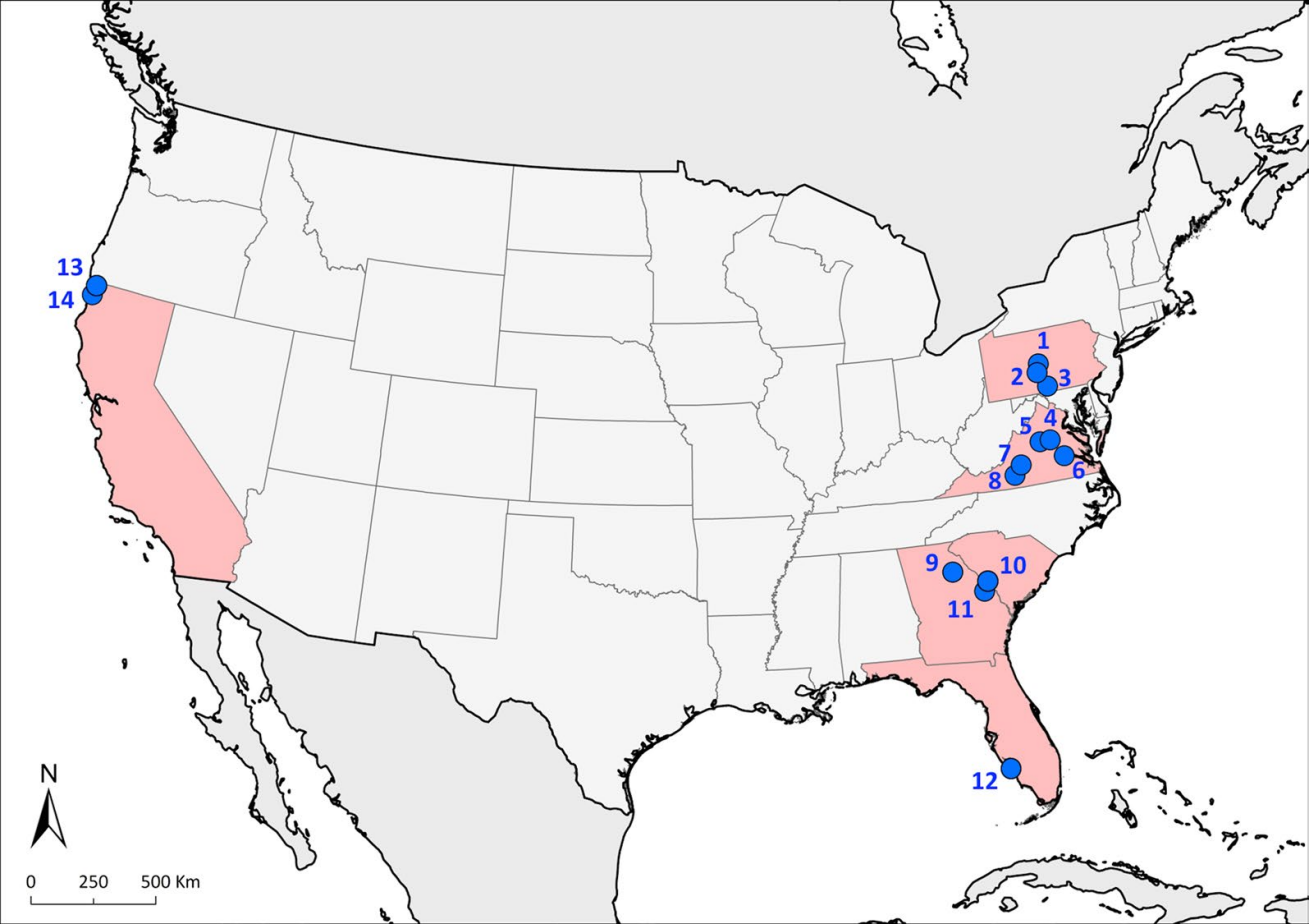
Geographical distribution of study locations in the United States. Study sites (blue circles): (1) Centre County, Pennsylvania, (2) Huntingdon County, Pennsylvania, (3) Franklin County, Pennsylvania, (4) Louisa County, Virginia, (5) Albermale County, Virginia, (6) Chesterfield County, Virginia, (7) Bedford County, Virginia, (8) Franklin County, Virginia, (9) Athens-Clarke County landfill, Georgia, (10) Savannah River Site, South Carolina, (11) Burke County, Georgia, (12) Lee County, Florida, (13) Del Norte County, California, (14) Humboldt County, California

Blood and/or tissue samples were opportunistically obtained from 162 TUVU and 95 BLVU in six U.S. states (Fig. 1, Table 2). In South Carolina, TUVU and BLVU were captured live at the Savannah River Site (33°20′39″ N 81°44′28″ W) and in Georgia, BLVU were captured live at the Athens-Clarke County landfill (33°54′57″ N 83°16′20″ W). Details of trapping methods used at these sites are described elsewhere [18]; briefly, carcass bait was placed within 25–50 m of a forest edge or roost to attract vultures to the site and birds were captured with a cannon net trigged manually from a blind. In California, TUVU were captured live as part of an on-going toxicological study at Humboldt County (41°08′07″ N 123°51′32″ W, 40°57′55″ N 124°02′38″ W, 40°51′31″ N 123°59′07″ W, and 40°43′02″ N 123°57′13″ W) and Del Norte County (41°59′07″ N 123°44′23″ W, 41°56′55″ N 124°06′51″ W, and 41°18′08″ N 124°03′17″ W); trapping involved use of both baited cannon net sets and walk-in/funnel trap described in detail elsewhere [19].

**Table 2:** Results of haemosporidian detection on New World vultures from the United States

Species	State	Context	Year	Sample type	Blood smears	PCR
Turkey vulture (TUVU)	South Carolina	Bait trapping	2013–2014	Blood	28/96 (29)	31/96 (32)
	California	Bait trapping	2010	Blood	NA	1/45 (2)
	Georgia	Nuisance removals	2016	Blood, muscle, and spleen	NA	2/4 (50)
	Virginia	Rehabilitation center	2016	Blood	NA	1/2 (50)
	Virginia	Bait trapping	2011–2012	Liver	NA	2/10 (20)
	Florida	Rehabilitation center	2016	Blood	0/2	2/2 (100)
	Pennsylvania	Carcasses	2016	Blood, muscle and spleen	NA	1/3 (33)
Black vulture (BLVU)	South Carolina	Bait trapping	2013–2014	Blood	0/79	0/79
	Georgia	Bait trapping	2013	Blood	0/11	0/11
	Georgia	Nuisance removals	2016	Blood, muscle, spleen	NA	0/3
	Virginia	Rehabilitation center	2016	Blood	NA	0/2

Diagnostic results are shown as: number of samples positive/number of samples tested (%)

*NA* not available

In Georgia, additional four TUVU and three BLVU samples were obtained from nuisance removals in Burke County (33°04′59″ N 82°01′19″ W). In Virginia, samples were obtained from two injured TUVU admitted for rehabilitation (Albemarle and Franklin counties) (38°01′43″ N 78°29′06″ W and 37°00′02″ N 79°53′05″ W) and from 10 TUVU sampled during a study on lead exposure in Chesterfield county (37°22′35″ N 77°30′23″ W). Additionally, two BLVU from Bedford and Louisa counties admitted for rehabilitation were sampled in Virginia (37°19′53″ N 79°31′23″ W and 38°01′31″ N 78°00′11″ W). In Florida, samples from two TUVU were obtained from birds from Lee County admitted for rehabilitation (26°38′35″ N 81°52′07″ W and 26°33′52″ N 81°57′53″ W). In Pennsylvania, samples were collected from three TUVU found dead in Franklin, Centre and Huntingdon counties (39°55′58″ N 77°39′05″ W, 40°46′30″ N 77°52′51″ W, and 40°28′39″ N 78°01′05″ W, respectively) that were submitted for diagnostic evaluation. Collection of the remainder of these samples were reviewed and approved by UGA’s IACUC protocol A2014 10-018.

When possible, blood samples were collected from the metatarsal vein. Two thin blood smears were immediately prepared in the field, air dried, fixed in 100% methanol, and later taken back to the laboratory to be stained with a modified Giemsa (DipQuick, Jorgensen Laboratories, Inc., Loveland, CO, USA). Remaining blood from most sites was preserved in heparin and frozen until PCR analysis. Blood samples from TUVU from California were placed on filter paper, dried and stored in a desiccant until testing. For deceased vultures, clotted blood, muscle, and spleen samples were obtained and frozen for PCR analysis. See Table 2 for which samples were collected from the different groups of birds.

### Parasite screening

Blood smears were examined at 1000× under oil immersion to determine infection status for blood parasites, with a minimum of 20,000 examined erythrocytes. Parasites were morphologically identified based on descriptions in the literature [2, 11, 16, 20]. Parasitaemia (no. parasites/erythrocytes examined) and the following morphometric parameters were obtained for a subset of mature gametocytes [2]: length and width of the parasite, length and width of infected and uninfected erythrocytes, number of pigment granules in the parasite, position of the parasite within the erythrocyte, and the ratio of nuclear displacement.

All tissue and/or blood samples were tested for *Haemoproteus* and *Plasmodium* using nested polymerase chain reaction (PCR). DNA was extracted from 10 µL of blood or ~ 10 mg of tissue using a commercial kit per the manufacturer’s instructions (Qiagen Dneasy Blood & Tissue Kit, Germantown, MD, USA). Nested PCR targeting the mitochondrial cytochrome b (*cytb*) gene was conducted as described using primary primers HaemNFI and HaemNR3 and nested primers HaemF and HaemR2 [21]. A subset of TUVU samples from Virginia (n = 12), Georgia (n = 3), Pennsylvania (n = 3) and Florida (n = 2) were tested for *Leucocytozoon* sp. using nested PCR using primary primers HaemNFI and HaemNR3 and nested primers HaemFL and HaemR2L [21]. Amplification products were visualized in 2% agarose gels stained with GelRed (Biotium, Hayward, CA, USA).

Amplification products of 18 TUVU samples (South Carolina = 11, Virginia = 3, Florida = 2, Pennsylvania = 1, California = 1) were extracted from the gel, purified using the QIAquick gel extraction kit (Qiagen), and submitted for bi-directional sequencing (using the HaemF and HaemR2 primers; 479 bp fragment) at the Georgia Genomics Facility (Athens, GA, USA). For one TUVU sample from South Carolina, a longer region of the *cytb* gene (725 bp) was sequenced using primers DW2 and DW4 [22]. For two TUVU samples from South Carolina, the nuclear adenylosuccinate lyase (*asl*) gene was sequenced using the primers described by Martinsen et al. [23]. Sequences obtained in this study were deposited in GenBank (accession numbers MF953291–MF953293).

### Phylogenetic analysis

Phylogenetic analysis of the *cytb* gene sequences was conducted to compare sequences obtained in this study to those of avian haemosporidians from the MalAvi database [24], as well as publicly-available sequences of reptilian and mammalian *Haemosporida* from GenBank (Additional file 1). Sequences were aligned using ClustalW [25] as implemented in MEGA 6.06 [26]. Bayesian phylogenetic trees were produced using MrBayes 3.2.6 [27]; the GTR + I + Γ model of nucleotide evolution was used as recommended by jModelTest 2.1.10 [28]. Two Markov chains were run simultaneously for 10 million generations with sampling every 1000 generations; the first 2500 trees (25%) were discarded as a burn-in step and the remaining trees were used to calculate the posterior probabilities.

### Statistical analyses

Comparison of prevalence based on PCR testing between vulture species, sampling sites, sampling method (live capture and nuisance removals vs. those that were in rehabilitation or were found dead), months and years was conducted using Fisher’s exact tests to account for low samples sizes for some groups. Variation of parasitaemia was compared in relation to age group (as determined in [17]), year and month of sampling, and sampling method was tested using Student’s T test or analysis of variance (ANOVA). Significance level was 0.05.

## Results

No parasites were detected by blood smears or PCR testing in BLVU samples. A total of 39/161 (24%) TUVU from six states were positive for haemosporidians by blood smear analysis and/or PCR testing (Table 2). All TUVU tested for *Leucocytozoon* spp. were PCR negative. The only other blood parasite observed in blood smears were microfilariae detected in two TUVU from South Carolina.

**Fig. 2:**
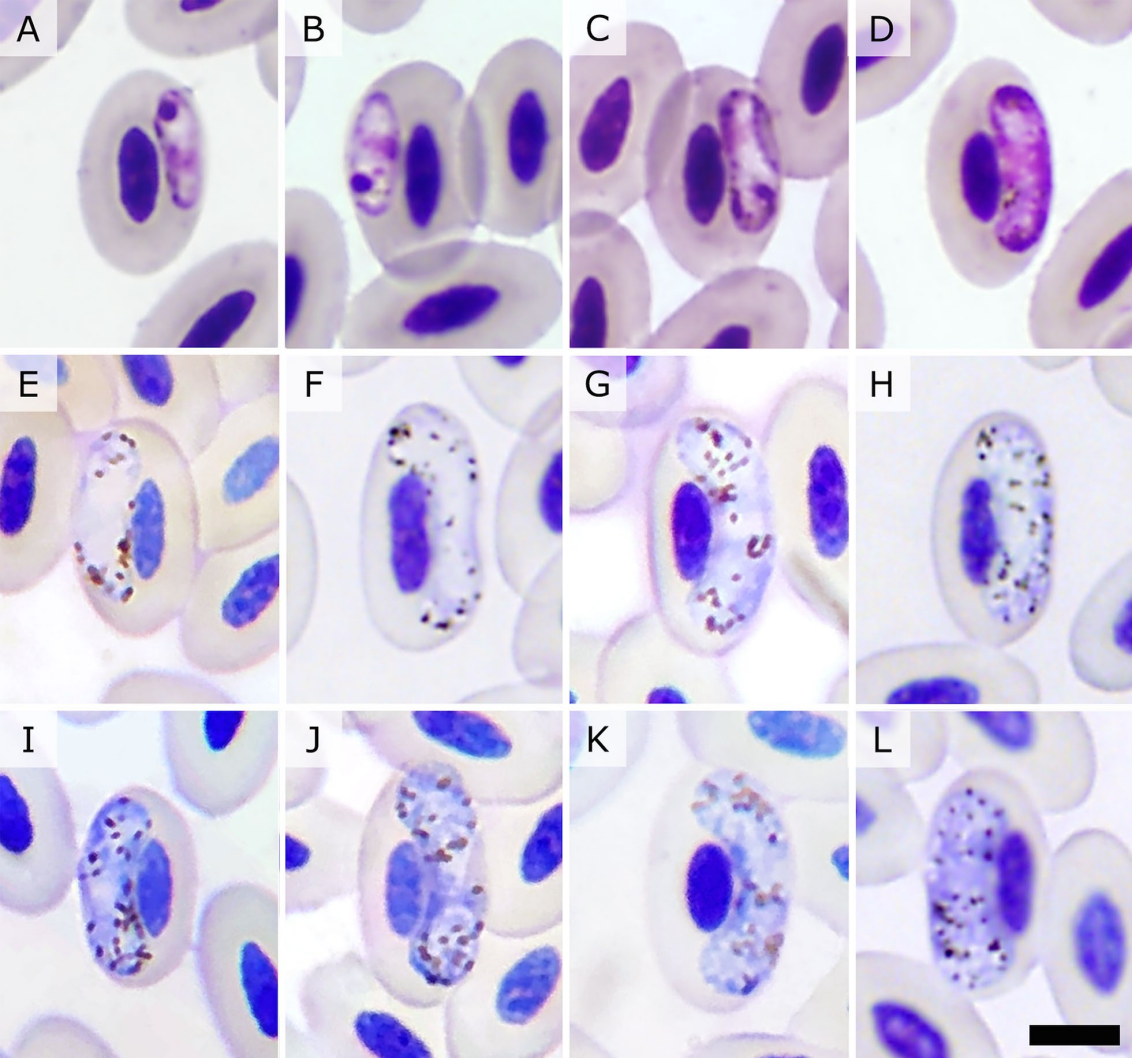
Photographs of *Haemoproteus catharti* in modified Giemsa-stained blood smears of turkey vultures (*Cathartes aura*) sampled at Savannah River Site, South Carolina, USA. **A**–**D** immature gametocytes, **E**–**H** microgametocytes, **I**–**L** macrogametocytes. Bar = 5 µm

All haemosporidian parasites observed in blood smears of TUVU from the South Carolina site were morphologically consistent with *H. catharti* as described for the same host and study site by Greiner et al. [16]. Immature gametocytes (Fig. 2A–D) are elongated and develop on lateral or subpolar position, without contact with the host cell nucleus, with pigment granules generally grouped near one of the poles of the parasite. Gametocytes (Fig. 2E–L) are thick, halteridial, with complete margins, with a centrally-located nucleus, with randomly or peripherally scattered pigment granules. Quantitative morphological parameters of *H. catharti* in this study were comparable to those reported by Greiner et al. [16]; however, in this study macrogametocytes and microgametocytes were slightly shorter and infected blood cells were slightly shorter in length, but wider (Table 3). Uninfected erythrocytes in this study (n = 35) had length (14.2 ± 0.7 μm, range 13–16 μm) and width (8.0 ± 0.6 μm, range 7–9 μm) similar to those reported by Greiner et al. [16] (respectively: 14.2 ± 0.8 μm, range 13–16 μm; 7.9 ± 0.5 μm, range 7–9 μm; n = 10).

**Table 3:** Morphometrics of *Haemoproteus catharti* as described by Greiner et al. [16] and in this study

	Macrogametocytes		Microgametocytes	
	This study	Greiner et al. [16]	This study	Greiner et al. [16]
	(n = 35)	(n = 20)	(n = 43)	(n = 3)
Gametocyte length (μm)	14.88 ± 1.60 [11–17]	15.4 ± 1.1 [14–18]	13.61 ± 1.30 [11–16]	14.5 ± 0.6 [14–15]
Gametocyte width (μm)	3.6 ± 0.5 [2.5–5.0]	3.4 ± 0.5 [2.5–5.0]	3.77 ± 0.70 [2.0–5.0]	3.7 ± 0.6 [3–4]
Nuclear displacement ratio (NDR)	0.57 ± 0.20 [0.22–1.00]	0.59 ± 0.2 [0.2–1.0]	0.46 ± 0.10 [0.2–0.7]	0.5 ± 0.2 [0.3–0.7]
Infected erythrocyte length (μm)	14.6 ± 0.7 [13–16]	15.4 ± 1.0 [14–17]	14.3 ± 0.9 [13–16]	15.2 ± 1.3 [14.0–16.5]
Infected erythrocyte width (μm)	8.1 ± 0.8 [7–10]	7.7 ± 0.7 [7–9]	8.2 ± 1.0 [6.0–10.5]	7.8 ± 0.8 [7.0–8.5]
Pigment granule number	25.6 ± 6.7 [9–38]	24.4 ± 7.0 [19–33]	22 ± 5.4 [12–34]	18.0 ± 7.0 [11–25]
Parasite in contact with host cell nucleus?	55% yes, 45% no	“Not usually”	49% yes, 51% no	“Not usually”

Values are shown as: mean ± SD [minimum–maximum]

The apparent prevalence of *H. catharti* in TUVU was significantly higher in the five eastern states (38/116, 33%) compared to the western state of California (1/45, 2%) (p < 0.001). At the South Carolina study site, the apparent prevalence of *H. catharti* was higher in 2013 (16/38, 42%) compared to 2014 (12/58, 21%) (p = 0.038) but no difference was noted between month of sampling (April 0/1 (0%) positive, May 10/27 (37%), June 11/42 (21%), and July 7/15 (47%); p > 0.05). For the TUVU sampled in the eastern United States, there was no difference in prevalence by sampling method (live capture/nuisance 35/109, 32% vs. sick or dead birds 4/7, 57%). In blood smear-positive birds (n = 28), parasitaemia was generally low (0.0302 ± 0.0326 parasites/erythrocyte, range 0.0049–0.1094) and was not significantly different in relation to age group (3 juveniles, 25 adults; p = 0.775), year (16 birds sampled in 2013, 12 in 2014; p = 0.369) or month of sampling (10 birds positive in May, 11 in June, 7 in July; p = 0.358).

**Fig. 3:**
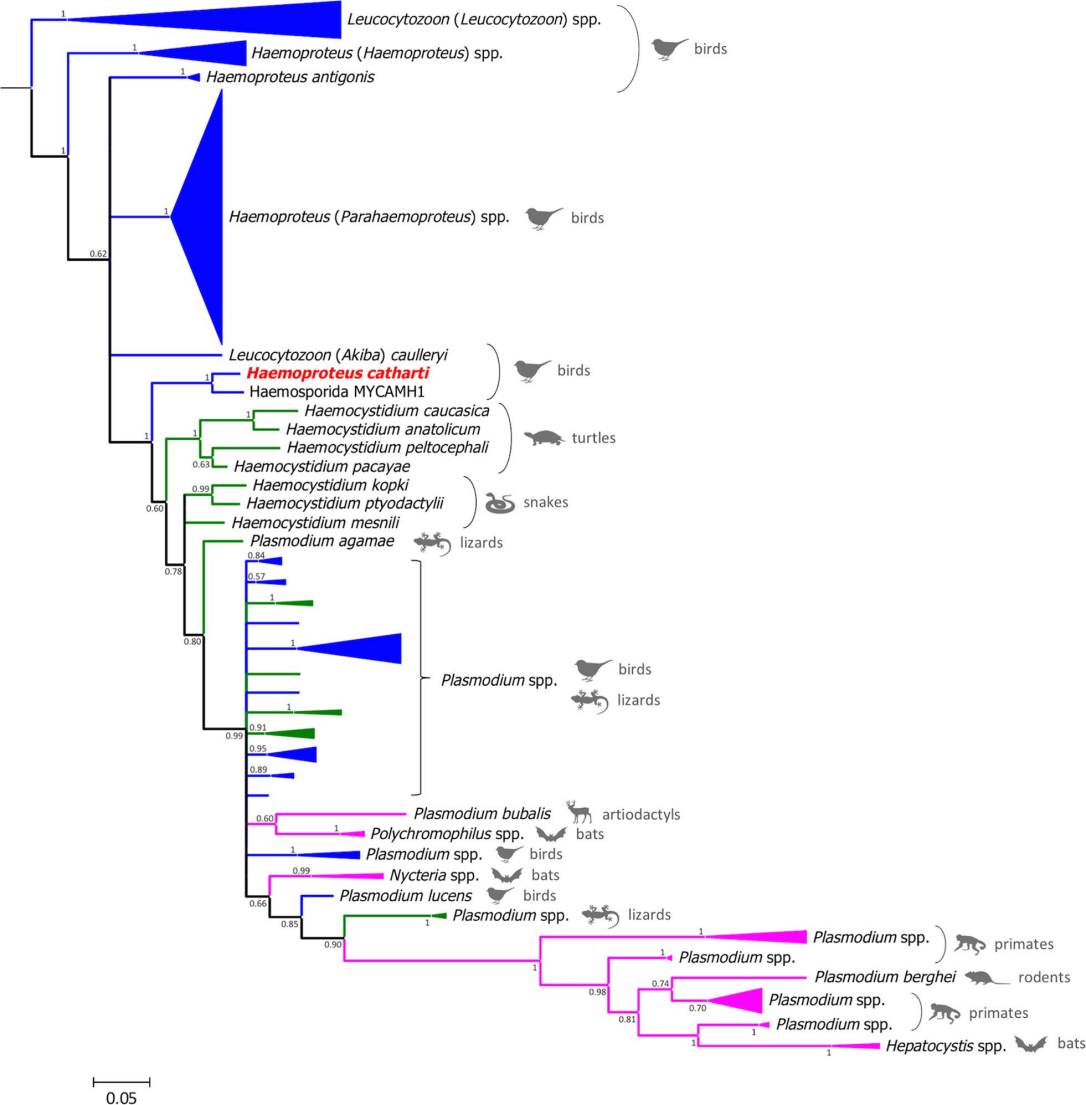
Bayesian phylogenetic tree of the mitochondrial cytochrome b gene sequences (479 bp) of the studied haemosporidian lineages. Sequences obtained in this study are emphasized in *red*. Branch lengths are drawn proportionally to evolutionary distance, and branches are coloured according to the host taxonomy: birds (blue), reptiles (green) and mammals (pink)

Partial *cytb* gene sequences (479 bp) of *H. catharti* from 17 TUVU from South Carolina, Virginia, Florida, and Pennsylvania were identical and differed from the California sequence by 1 bp (99.8% identity). The *cytb* gene sequences of *H. catharti* were most similar (97.1% identity, 481 bp) to that of *Haemosporida* lineage MYCAMH1 (GenBank accession code JX546141). Bayesian phylogenetic trees produced for the *cytb* gene sequences are shown in Fig. 3; more detailed versions of these trees are provided in Additional file 1. These analyses indicate that *H. catharti* and MYCAMH1 grouped in a clade that was distinct from other *Haemoproteus* spp. and suggested a close relationship with *Haemocystidium* and *Plasmodium* spp.

Partial *asl* sequences (246 bp) from two TUVU from the South Carolina site were identical. These sequences were nearly equally similar to several avian, mammalian, and reptilian representatives in genera such as *Polychromophilus* (80–83%), *Nycteria* (79–84%), and *Plasmodium* (79–83%). However, the *asl* gene sequences had much lower identity to the other two avian malaria genera *Haemoproteus* (76–80%) and *Leucocytozoon* (71–79%).

## Discussion

*Haemoproteus catharti* was detected in TUVU at all studied US states, but no evidence of infection was detected in BLVU by either PCR assay or blood smear analysis. This difference is surprising considering that these species have a shared evolutionary history [29] and have extensive similarities in the functional ecology, as well as in morphological, physiological, and behavioural attributes [9, 10]. The closest relatives of TUVU are endemic to South America, the lesser yellow-headed vulture (*Cathartes burrovianus*) and the greater yellow-headed vulture (*Cathartes melambrotus*) [8]. Neither of these species have been examined for blood parasites, thus future studies would be valuable to determine if these species have parasites related to *H. catharti*. The only haemoparasites detected in the current study were *H. catharti* and unidentified microfilaria, but paired blood smears and blood for PCR testing were only available from one site so it is possible that other parasites were not detected as the PCR assay used will amplify both *Plasmodium* and *Haemoproteus* and lack of amplification of some parasite species with commonly used PCR assays has been reported [5].

A single nucleotide difference in the *cytb* gene region was detected in the sequences of *H. catharti* detected in the two subspecies of TUVU sampled, *Cathartes aura septentrionalis* from the eastern United States and *Cathartes aura meridionalis* in California. These two TUVU subspecies do not overlap in residential range and have distinct migration patterns with *C. a. septentrionalis* being much less migratory than *C. a. meridionalis* [30, 31]. It is unknown if this genetic difference is due to TUVU subspecies isolation or general geographic variation. In the 1960s, Galindo and Sousa [32] reported *Haemoproteus* in TUVU from Panama, which could possibly be in *Cathartes aura ruficolis*. However, the authors noted that the birds sampled were migrants flying through so it is unknown if infections were actually in migrating *C. a. meridionalis* from the western United States. Future molecular characterization studies of additional *H. catharti* samples from the different TUVU subspecies are needed to determine if the parasites vary by subspecies or geographic location.

The phylogenetic analysis of *cytb* gene sequences revealed that *H. catharti* is closely related to *Haemosporida* sp. MYCAMH1, a yet unidentified parasite of wood storks (*Mycteria americana*) in southeastern USA (Georgia state) and northern Brazil (Amapá state) [33]. Initially MYCAMH1 had been classified as a *Haemoproteus* sp. and although the morphology of this lineage has not been described, Villar et al. [33] provided a photomicrograph of a gametocyte that shows the parasite contains pigmented granules and thus generally conforms to *Haemoproteus*. However, *H. catharti* and MYCAMH1 constitute a clade that is unmistakably separate from all other *Haemoproteus* spp., being most closely related to *Haemocystidium* spp. from reptiles and to *Plasmodium* spp. from birds and reptiles. The partial *asl* gene sequence obtained in the current study also suggests that *H. catharti* is clearly distinct from all other *Haemoproteus* spp., being instead most similar to other haemosporidian genera, such as *Polychromophilus*, *Nycteria* and *Plasmodium*.

Based on their natural history and morphological characteristics, pigmented haemosporidian that infect avian erythrocytes without forming erythrocytic meronts would traditionally be placed in the genus *Haemoproteus* [2]. However, the genetic evidence produced in this study suggests this parasite (along with MYCAMH1) might represent a novel genus. This would place *H. catharti* in an analogous condition to *Haemoproteus antigonis*, which was recently discovered to represent a separate clade from the remainder *Haemoproteus* spp. [5]. Thus, it is clear that a taxonomic revision of avian haemosporidians is warranted, possibly with the designation of novel, separate, genera for *H. antigonis* and *H. catharti*. It is worth noting that, based on the presence of pigment in gametocytes and the absence of merogony in blood cells, *H. antigonis* and *H. catharti* would still be classified in the family Haemoproteidae alongside with other genera such as *Haemocystidium*, *Haemoproteus*, *Hepatocystis,* and *Polychromophilus* [34].

There are a number of records of *Haemoproteus* sp. in BLVU and TUVU for which the parasites were not morphologically or genetically characterized (Table 1); it is reasonable to suspect that some of these records—or perhaps all of them—correspond to *H. catharti*. Neither of the two species of *Haemoproteus* recorded in Old World vultures, *H. elani* and *H. janovyi*, have been molecularly characterized; therefore, it is not possible at present to evaluate their phylogenetic relationship to *H. catharti*. Of these, *H. elani* bears remarkable morphological similarities to *H. catharti*, as in both species: (a) fully grown gametocytes are halteridial and do not completely encircle the infected erythrocyte nucleus (Fig. 2E–L), (b) fully grown gametocytes have variable contact with the infected erythrocyte nucleus (Fig. 2E, I–K), and (c) fully grown gametocytes fill the infected erythrocytes up to their poles (Fig. 2G) [2, 16]. It is worth noting that *H. elani* is traditionally considered a parasite of hawks and eagles (Accipitriformes) [20] and there are only two records of this parasite in an Old World vultures: a lappet-faced vulture (*Torgos tracheliotos*) captive at the Oklahoma Zoo, USA [12] and a white-backed vulture (*Gyps africanus*) sampled at Nossob Camp, Cape of Good Hope, Western Cape, South Africa (IRCAH accession number 103937—M. Bryant, pers. comm.) [35]. Considering the morphological similarities between *H. elani* and *H. catharti*, future studies would benefit from evaluating the gene sequences of these parasites to appraise their evolutionary relationship and their relation to *H. elani* strains from hawks and eagles.

It is interesting to note that the phylogenetic proximity between *H. catharti* and MYCAMH1 seems to parallel early, and apparently incorrect, suggestions of a close relationship between New World vultures (Cathartiidae) and storks (Ciconiidae) [36, 37]. In recent years, however, it has been proposed that New World vultures and storks have a paraphyletic origin and Cathartiidae should be placed in its own order, Cathartiformes [38], being most closely related to hawks and eagles [39]. Future studies on the molecular biology of haemosporidian parasites from Accipitridae, Cathartiidae and Ciconiidae would thus be valuable in clarifying the host specificity of these organisms and their transmission and evolution across host taxonomic boundaries.

Lastly, if *H. catharti* and MYCAMH1 are not closely related to other *Haemoproteus* spp., it is possible that different vectors are involved in their transmission. *Haemoproteus* (*Parahaemoproteus*) spp. and *Leucocytozoon caulleryi* are transmitted by Ceratopogonidae (biting midges), *Haemoproteus* (*Haemoproteus*) spp. are transmitted by Hippoboscidae (louse flies), *Plasmodium* spp. are transmitted by Culicidae (mosquitoes), and *Leucocytozoon* (*Leucocytozoon*) spp. are transmitted by Simuliidae (black flies) [2]. The vectors of the reptile-infecting *Haemocystidium* spp. (formerly classified as *Haemoproteus*) are poorly understood but one *Haemocystidium* species, *H. metchnikovi*, has been successfully transmitted to painted turtles (*Chrysemys picta*) by Tabanidae (horse flies) [40]. The vector(s) of *Fallisia neotropicalis* are unknown but there are some experimental data that suggest mosquitoes may be vectors [2]. It is, therefore, reasonable to consider these families of dipteran insects as potential candidates to be the vectors of *H. catharti* (and likely MYCAMH1).

## Conclusions

*Haemoproteus catharti* is a widely distributed parasite of TUVU in North America that is evolutionarily distinct from other haemosporidian parasites. These data, alongside those of a recent study on the haemosporidian parasites of North American cranes [5], reveal that the genetic diversity and evolutionary relationships of avian haemosporidians are still being uncovered. Future studies combining a comprehensive evaluation of morphological and life cycle characteristics with the analysis of multiple nuclear and mitochondrial genes are needed to redefine the genus boundaries of these parasites and to re-evaluate the relationships amongst haemosporidians of birds, reptiles and mammals.

## Additional file

**Additional file 1.** Bayesian phylogenetic tree for select reptilian, avian and mammalian haemosporidians based on mitochondrial cytochrome b gene sequences. Branch lengths are drawn proportionally to evolutionary distance and posterior probability values are shown. GenBank or MalAvi ascension codes are provided for each sequence. Branches are colour coded by parasite host; avian species are blue, reptile hosts are green, and mammal hosts are pink.
