# Supplementary figures and images for: Parasitaemia data and molecular characterization of Haemoproteus catharti from New World vultures (Cathartidae) reveals a novel clade of Haemosporida
Source: Malar J. 2018 Jan 8;17:12. doi: 10.1186/s12936-017-2165-5 (PMC5759834; doi:10.1186/s12936-017-2165-5)

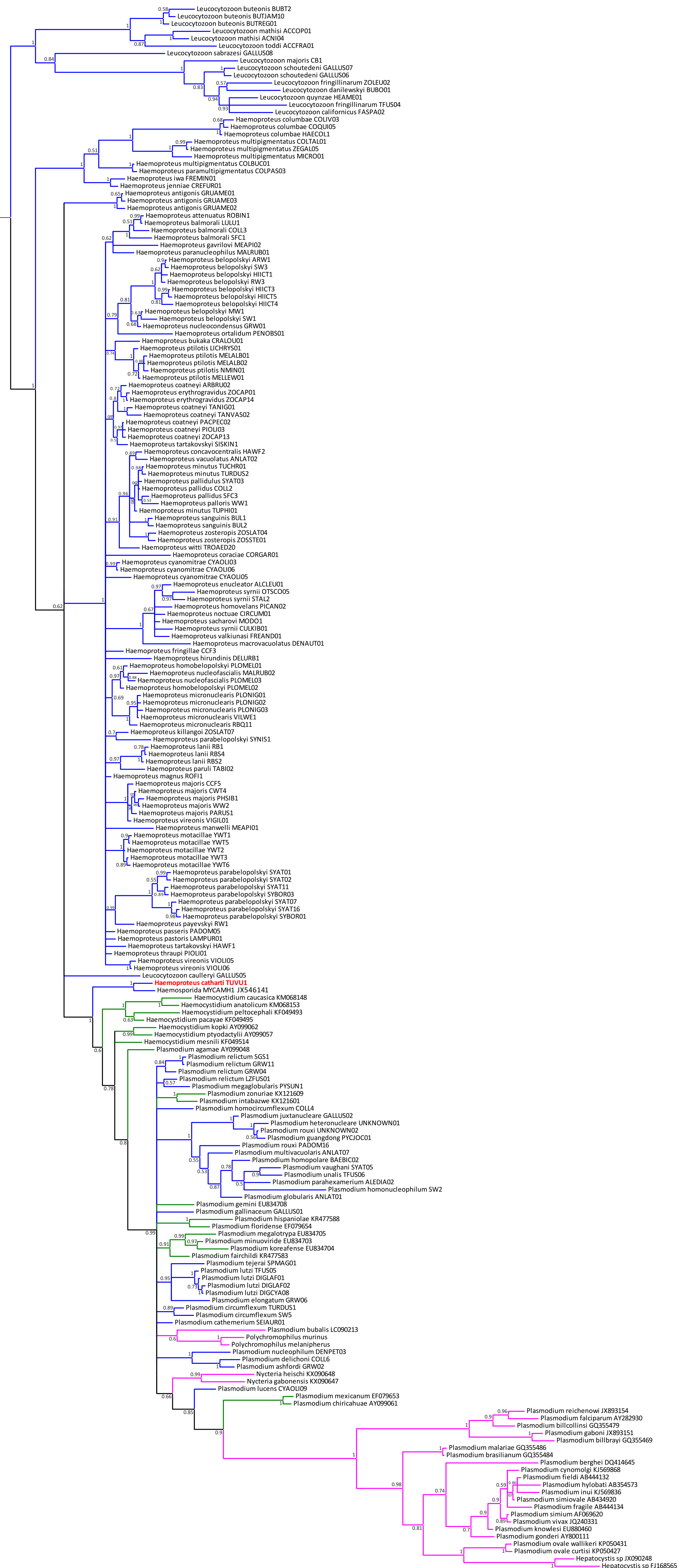

0.05

Supplement: Supplementary file 1 — Additional file 1. Bayesian phylogenetic tree for select reptilian, avian and mammalian haemosporidians based on mitochondrial cytochrome b gene sequences. Branch lengths are drawn proportionally to evolutionary distance and posterior probability values are shown. GenBank or MalAvi ascension codes are provided for each sequence. Branches are colour coded by parasite host; avian species are blue, reptile hosts are green, and mammal hosts are pink. [file 12936_2017_2165_MOESM1_ESM.pdf]
